# Association between cannabis use disorder and schizophrenia stronger in young males than in females

**DOI:** 10.1017/S0033291723000880

**Published:** 2023-11

**Authors:** Carsten Hjorthøj, Wilson Compton, Marie Starzer, Dorte Nordholm, Emily Einstein, Annette Erlangsen, Merete Nordentoft, Nora D. Volkow, Beth Han

**Affiliations:** 1 Copenhagen Research Center for Mental Health – CORE, Mental Health Center Copenhagen, Copenhagen University Hospital, Copenhagen, Denmark; 2 Department of Public Health, University of Copenhagen, Section of Epidemiology, Copenhagen, Denmark; 3 National Institute on Drug Abuse, National Institutes of Health, Bethesda, USA; 4 Danish Research Institute for Suicide Prevention, Mental Health Centre Copenhagen, Copenhagen, Denmark; 5 Department of Mental Health, Johns Hopkins Bloomberg School of Public Health, Baltimore, MD, USA; 6 Centre for Mental Health Research, Research School of Population Health, The Australian National University, Canberra, Australia; 7 Department of Clinical Medicine, University of Copenhagen, Faculty of Health and Medical Sciences, Copenhagen, Denmark

**Keywords:** Cannabis, epidemiology, gender, psychosis, schizophrenia, sex differences, sex

## Abstract

**Background:**

Previous research suggests an increase in schizophrenia population attributable risk fraction (PARF) for cannabis use disorder (CUD). However, sex and age variations in CUD and schizophrenia suggest the importance of examining differences in PARFs in sex and age subgroups.

**Methods:**

We conducted a nationwide Danish register-based cohort study including all individuals aged 16–49 at some point during 1972–2021. CUD and schizophrenia status was obtained from the registers. Hazard ratios (HR), incidence risk ratios (IRR), and PARFs were estimated. Joinpoint analyses were applied to sex-specific PARFs.

**Results:**

We examined 6 907 859 individuals with 45 327 cases of incident schizophrenia during follow-up across 129 521 260 person-years. The overall adjusted HR (aHR) for CUD on schizophrenia was slightly higher among males (aHR = 2.42, 95% CI 2.33–2.52) than females (aHR = 2.02, 95% CI 1.89–2.17); however, among 16–20-year-olds, the adjusted IRR (aIRR) for males was more than twice that for females (males: aIRR = 3.84, 95% CI 3.43–4.29; females: aIRR = 1.81, 95% CI 1.53–2.15). During 1972–2021, the annual average percentage change in PARFs for CUD in schizophrenia incidence was 4.8 among males (95% CI 4.3–5.3; *p* < 0.0001) and 3.2 among females (95% CI 2.5–3.8; *p* < 0.0001). In 2021, among males, PARF was 15%; among females, it was around 4%.

**Conclusions:**

Young males might be particularly susceptible to the effects of cannabis on schizophrenia. At a population level, assuming causality, one-fifth of cases of schizophrenia among young males might be prevented by averting CUD. Results highlight the importance of early detection and treatment of CUD and policy decisions regarding cannabis use and access, particularly for 16–25-year-olds.

## Introduction

Cannabis is among the most frequently used psychoactive substance in the world, and laws restricting cannabis use have been liberalized over the past 20 years (Compton, Han, Jones, Blanco, & Hughes, 2016). Based on the World Health Organization's 2021 World Drug Report, approximately 200 million people in the world used cannabis in 2019 (UNODC, 2021). Moreover, cannabis potency measured by the percentage of delta-9-tetrahydrocannabinol (THC) (main psychoactive component of cannabis) has increased dramatically, e.g. from −10% in 2009 to 14% in 2019 in the USA (ElSohly, Chandra, Radwan, Majumdar, & Church, 2021); and from 13% in 2006 to 30% in 2016 in Denmark (Freeman et al., 2019). Consistently, the prevalence of cannabis use disorder (CUD) has increased markedly. For example, past-year CUD rose significantly from 4.9% in 2014 to 5.9% in 2018 among US 18–25-year-olds.

THC may trigger and/or worsen schizophrenia, especially for those with a CUD or with regular and high THC use (Marconi, Di Forti, Lewis, Murray, & Vassos, 2016; Petrilli et al., 2022; Volkow et al., 2016). For example, in Denmark, the incidence of schizophrenia steadily increased from 2000 to 2012 (Kühl, Laursen, Thorup, & Nordentoft, 2016), and the schizophrenia population attributable risk fraction (PARF) for CUD increased three- to fourfold over the past two decades, parallel to increases in THC concentration (Hjorthøj, Posselt, & Nordentoft, 2021). The increased THC content may thus, along a potential increase in the prevalence of CUD, be a main driver of the population-level increase in PARF between CUD and schizophrenia.

A growing body of evidence suggests that the relationship between CUD and schizophrenia may differ by sex (Arranz et al., 2015; Crocker & Tibbo, 2018). Male sex (Arranz et al., 2015; Veen et al., 2004) and early heavy or frequent cannabis use are associated with earlier onset of psychosis (Han, Compton, Einstein, & Volkow, 2021; Large, Sharma, Compton, Slade, & Nielssen, 2011). Although it has not been shown that there are sex differences in age of first cannabis use (Crane, Schuster, Mermelstein, & Gonzalez, 2015; SAMHSA, 2019), younger age of CUD onset was found in males compared to females (Haberstick et al., 2014). Past-year prevalence of daily or near daily cannabis use and CUD were consistently higher in males than females among US adults aged 18–34 in each year during 2008–2019 (Han et al., 2021). The same was seen in Denmark where schizophrenia incidence rates were consistently higher in males than females among patients aged 19 or older in each year during 2000–2012 (Kühl et al., 2016).

It has been proposed that the higher incidence of schizophrenia among males than females could reflect the higher prevalence and quantity of consumption of cannabis use in males (Ochoa, Usall, Cobo, Labad, & Kulkarni, 2012; Sommer, Tiihonen, van Mourik, Tanskanen, & Taipale, 2020). Consequently, it is pivotal to understand whether and how incidence of schizophrenia attributable to CUD varies by sex and age.

Thus, based on nationwide Danish registers, this current study aimed to investigate:

1. Do the associations between CUD and schizophrenia vary by sex?
2. Do the sex differences in the associations between CUD and schizophrenia change over time and by age?
3. Does the proportion of schizophrenia cases attributable to CUD vary by sex?
4. Does the sex-specific proportion of schizophrenia cases attributable to CUD change over time and by age?

The results of this study may inform ongoing policy discussions on legalization and regulation of cannabis use and highlight the importance of targeted public health prevention and intervention efforts. Because males, especially those with CUD, often have worse schizophrenia treatment outcomes than their female counterparts (Abel, Drake, & Goldstein, 2010; Arranz et al., 2015), our results may also have implications beyond policy, underscoring the need for clinicians to proactively screen for and diagnose CUD and schizophrenia and deliver sex-specific, high-quality, and patient-centered care.

## Methods

### Data sources

We used the nationwide Danish registers, full-linkage of which is made possible through the unique identification number in the Civil Registration System (Pedersen, 2011). We included all people born before 31 December 2005, and who were alive and aged between 16 and 49 (both inclusive) at some point during 1972–2021.

### Cannabis use disorder and schizophrenia

Information on psychiatric disorders was obtained from the Psychiatric Central Research Register and the psychiatric section of the National Patient Register, which contains information on all psychiatric inpatient treatments in Denmark since 1969, supplemented with all outpatient treatments since 1995 (Lynge, Sandegaard, & Rebolj, 2011; Mors, Perto, & Mortensen, 2011). Within these registers, schizophrenia was defined as ICD-8 codes 295.X (except 295.7) and ICD-10 codes F20.X. CUD was identified in the same registers and supplemented with the somatic part of the National Patient Register, defined as ICD-8 code 304.5 and ICD-10 code F12.X.

### Other variables

We also included information on alcohol use disorder (AUD) and other types of substance use disorders, using the same registers as described above, using ICD-8 codes 291.X, 303.X, 571.0, and 304.X (except 304.5) and ICD-10 codes F1X.X (except F12.X, E24.4, E52, G31.2, G62.1, G72.1, K29.2, K70, K86.0, O35.4, Y57.3, Z50.2, Z50.3, Z71.4, Z71.5, Z72.1, and Z72.2). We included information on whether an individual had been diagnosed with any other psychiatric disorder (remaining ICD-8 codes from 290 to 315, and remaining ICD-10 codes in the F chapter). Sex was defined from the civil registration system registry, which will include the sex assigned at birth, except in the (in Denmark rather rare) cases in which a person legally changed their registered sex or gender in this registry. We also examined information on parental history of the same disorders and whether a person was Danish-born.

### Statistical analyses

First, we conducted Cox proportional hazards regression analyses (with age as the underlying time-variable) in the full population, treating alcohol and specific drug use disorders, including CUD, as time-varying covariates, and included an interaction term between CUD and sex. If this interaction term was statistically significant, stratified multivariable Cox proportional hazards regression analyses (adjusting for the aforementioned potential confounders) by sex were conducted. We estimated PARFs overall and for males and females, separately, using the formula pd×([HR–1]/HR), with pd being the prevalence of CUD among cases developing schizophrenia.

Next, to examine trends in adjusted PARFs within each sex, we estimated PARFs for each study year through applying the fully adjusted Cox proportional hazards regression models by year, for males and females separately. To test the assumption of proportional hazards, we visually inspected both plots of Schoenfeld residuals and log-minus-log plots, neither indicating important deviations from this assumption.

Based on adjusted PARF results for each study year, joinpoint Regression Program (version 4.8.01) was used to test for significant changes in trends using Bayesian information criterion and to estimate average annual percentage changes in PARFs of CUD in schizophrenia during 1972–2021, which are valid even if the joinpoint models indicate changes in trends during this study period. In particular, joinpoint Regression Program takes trend data and fits the simplest joinpoint model that the data allow, starting with the minimum number of joinpoints (e.g. 0 joinpoint, which is a straight line) and testing whether more joinpoints are statistically significant and must be added to the model (up to that maximum number). This enables us to test whether a change in trend is statistically significant. The tests of significance use the Bayesian information criterion, which finds the model with the best fit for the data.

Finally, we applied Poisson regression and evaluated the potential three-way interaction effect among CUD, sex, and age (as a time-varying covariate, coded as ages 16–20, 21–25, 26–30, 31–40, ⩾41) on the incidence of schizophrenia. The analyses allowed an interaction with the underlying time-scale (age). Except for Joinpoint regression, all other analyses were conducted in Stata/MP, version 16.1 (StataCorp LLC). For each analysis, *p* < 0.05 (two-tailed) was considered statistically significant.

## Results

This study examined 6 907 859 individuals and 45 327 cases of incident schizophrenia during a follow-up of 129 521 260 person-years at risk. Table 1 shows characteristics of the study population, and online Supplementary Fig. S1 shows the distribution of date of birth of the cohort. The unadjusted hazard ratio (HR) for people with CUD to be diagnosed with schizophrenia was 30.18 (95% CI 29.31–31.08; *p* < 0.001). However, after adjusting for potential confounding factors in the pooled model, this was reduced to an adjusted HR (aHR) = 2.31 (95% CI 2.24–2.40; *p* < 0.001), and a significant interaction effect between sex and CUD was identified (*p* < 0.001). Thus, stratified analyses by sex were conducted, showing that the overall aHR for CUD on schizophrenia was slightly higher (*p* < 0.001) among males (aHR = 2.42, 95% CI 2.33–2.52) than females (aHR = 2.02, 95% CI 1.89–2.17) (Table 2). Time since CUD and incident schizophrenia is depicted in online Supplementary Fig. S2.

**Table 1.** Characteristics of the study population overall and by sex, *N* (%)

	Overall	Males	Females
Male	3 531 266 (51.1%)	3 531 266 (100%)	–
Females	3 376 593 (48.9%)	–	3 376 593 (100%)
Foreign born	626 671 (9.1%)	315 723 (8.9%)	310 948 (9.2%)
Parental schizophrenia	30 103 (0.4%)	15 590 (0.4%)	14 513 (0.1%)
Other parental psychiatric disorder	835 738 (12.1%)	434 776 (12.3%)	400 962 (11.9%)
Parental alcohol or substance use disorder	639 845 (9.3%)	329 495 (9.3%)	310 350 (9.2%)
Cannabis use disorder	60 563 (0.9%)	45 322 (1.3%)	15 241 (0.5%)
Alcohol use disorder	398 430 (5.8%)	265 578 (7.5%)	132 852 (3.9%)
Other substance use disorder	233 512 (3.4%)	126 910 (3.6%)	106 602 (3.2%)
Other psychiatric disorder	869 274 (12.6%)	397 389 (11.3%)	471 885 (14.0%)

Data source: the nationwide Danish registers, full-linkage of which is made possible through the unique identification number in the Civil Registration System.

**Table 2.** Adjusted hazard ratios of cannabis use disorder CUD on schizophrenia by sex and adjusted incidence rate ratios of CUD on schizophrenia by sex and age group

Overall	No CUD	Males with CUD adjusted hazard ratio (95% CI)	Females with CUD adjusted hazard ratio (95% CI)	*P* value for the sex difference
	1 (ref.)	2.42 (2.33–2.52)	2.02 (1.89–2.17)	*p* < 0.001
By age group	No CUD	Males with CUD adjusted incident rate ratio (95% CI)	Females with CUD adjusted incident rate ratio (95% CI)	
16–20 years	1 (ref.)	3.84 (3.43–4.29)	1.81 (1.53–2.15)	*p* < 0.001
21–25 years	1 (ref.)	2.58 (2.38–2.79)	1.91 (1.27–1.64)	*p* = 0.02
26–30 years	1 (ref.)	2.33 (2.12–2.57)	2.08 (1.72–2.52)	*p* = 0.70
31–40 years	1 (ref.)	2.13 (1.94–2.34)	2.31 (1.92–2.78)	*p* = 0.91
41+ years	1 (ref.)	2.18 (1.87–2.54)	2.98 (2.32–3.84)	*p* = 0.16

Adjusted for alcohol use disorder (AUD), other substance use disorder (SUD), other psychiatric disorders, parental history of CUD, AUD, SUD, schizophrenia, or other psychiatric disorders, and whether a person was Danish-born.

**Figure 1.**
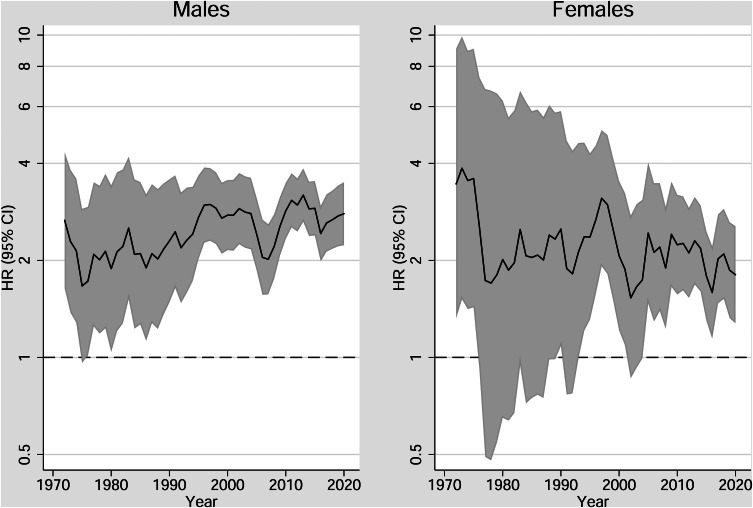
Rolling average of adjusted hazard ratios between cannabis use disorder and schizophrenia, by sex and calendar year.

Figure 1 shows the aHR for CUD on incident schizophrenia by year for males and females, separately. For males, the aHR increases gradually from ~2 to ~3, whereas for females, there is no such clear pattern. The corresponding aHR for AUD and other substance use disorder are shown in online Supplementary Figs S3 and S4, respectively. Note that for AUD, the estimates were not stable until 1994, which is consequently the first year included in online Supplementary Fig. S3. The lifetime prevalence of CUD for males and females is presented in online Supplementary Fig. S5.

**Figure 2.**
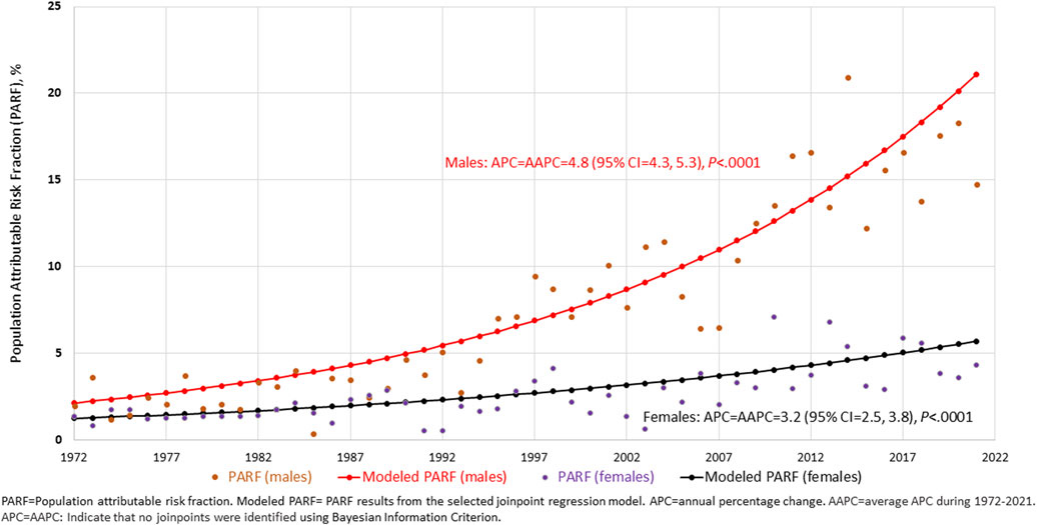
Trends in the proportion of schizophrenia attributable to cannabis use disorder in Denmark during 1972–2021, by sex.

Moreover, during 1972–2021, among males, the annual average percentage change in the PARFs of CUD on the incidence of schizophrenia was 4.8 (95% CI 4.3–5.3; *p* < 0.0001; no joinpoint identified); whereas among females, it was 3.2 (95% CI 2.5–3.8; *p* < 0.0001; no joinpoint identified) (Fig. 2). These results suggest that during 1972 throughout 2021, the annual average percentage change in the PARFs of CUD on the incidence of schizophrenia was consistently higher in males than in females (*p* < 0.0001). Assuming causality, approximately 15% of recent cases of schizophrenia among males in 2021 would have been prevented in the absence of CUD; by contrast, among females, 4% of recent cases of schizophrenia would have been prevented if they did not have CUD.

In the fully adjusted Poisson regression model, a significant three-way interaction among CUD, age, and sex was identified (*p* < 0.001). Table 2 shows the incidence rate ratio (IRR) and 95% CI for each of the 10 age–sex combinations. For males, the highest adjusted incidence rate ratio (aIRR) of CUD on schizophrenia was observed in 16–20-year-olds (aIRR = 3.84, 95% CI 3.43–4.29), which was more than twice as high (*p* < 0.001) than their female similar-age peers (aIRR = 1.81, 95% CI 1.53–2.15). For males aged 21–25, adjusted IRR was 2.58 (95% CI 2.38–2.79), which was 1.4 times higher (*p* = 0.02) than their female similar-age peers [females aged 21–25: aIRR = 1.91 (95% CI 1.27–1.64)]. The association between CUD and schizophrenia was not statistically different between males and females for those aged ⩾26.

**Figure 3.**
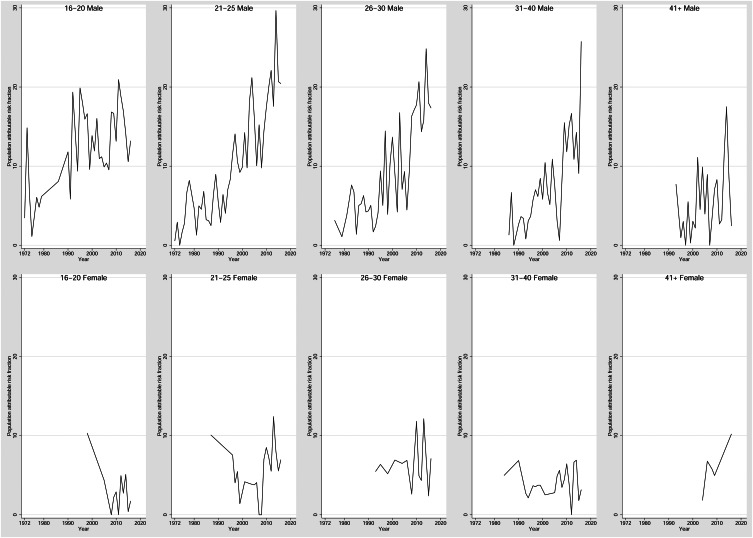
Trends in the proportion of schizophrenia attributable to cannabis use disorder in Denmark during 1972–2021, by sex and age.

Trends in PARFs for the 10 age–sex combinations show very different patterns (Fig. 3). Among 16–20-year-old males, no clear pattern over time is observed, with PARFs generally fluctuating between 10% and 20%. In older males, the PARFs show a clearly increasing pattern, ending up around 20–30% until the age of 31–40 when PARFs fluctuate between <1% and nearly 20%. For females, the PARFs were not always estimable due to low numbers of exposed cases, but no clear association with time was observed, and with very few exceptions, PARFs for females were 10% or lower.

## Discussion

In this nationwide, register-based cohort study, we found evidence of a stronger association between CUD and schizophrenia for males than for females, consistent with the results from a small clinical sample indicating that females experiencing CUD are at lower risk of developing psychosis than males (Arranz et al., 2015). Further, the stronger PARFs of CUD in schizophrenia for males than females consistently increased from 1972 to 2021. Under the assumption of causality, in 2021, approximately 15% of recent cases of schizophrenia among males would have been prevented in the absence of CUD, in contrast to 4% among females. For younger males, the proportion of preventable CUD-associated cases may be as high as 25% or even 30%. This increase in PARF is related to both increasing associations, likely caused by more potent cannabis, and increasing the prevalence of CUD with time.

The aHR for CUD on risk of schizophrenia were slightly higher for males than females, which might be misinterpreted to suggest an overall absence of strong sex-specific effects of cannabis, and instead indicate that the lower PARF for females is due to the fact that fewer females than males have CUD. Importantly, when subdividing the sample into specific age groups, a strong interaction effect among age, sex, and CUD on schizophrenia became evident. For 16–20-year-olds, the adjusted IRR for the association between CUD and schizophrenia was nearly twice as high for males than females; for 21–25-year-olds, the IRR was approximately 50% higher for males than females, whereas for those aged >26 years of age the IRRs was similar for males and females. Assuming causality, this suggests that young males compared to females of the same age might be more susceptible to the psychotogenic effects of cannabis on schizophrenia. Further research is needed to examine potential differences in THC concentration of exposures and frequency of cannabis consumption between young males and young females (Khan et al., 2013).

Previous studies have indicated that partial genetic confounding factors likely exist on the association between CUD and schizophrenia, i.e. genes shared between these conditions may account for some but not all of the association (Gillespie & Kendler, 2020). However, genetic confounding factors would be unlikely to explain the steeper increases in the PARFs of CUD on schizophrenia that we identified for males than females, as changes in the genetic risk-profile of an entire population would have to occur over generations. Both preclinical and clinical studies have provided evidence of significant differences between the sexes in response to the acute and long-term effects of cannabis (Cooper & Craft, 2018). We were able to adjust for alcohol and other specific drug use disorders in our study, but not for tobacco use or tobacco use disorder, which has also been linked to psychosis in some (Gurillo, Jauhar, Murray, & MacCabe, 2015) but not all studies (Fergusson, Hall, Boden, & Horwood, 2015). Thus, future research is needed to investigate the mechanisms underlying the higher vulnerability of young males to the effects of cannabis on schizophrenia than that of young females.

This study adds to the evidence suggesting a relationship between intense use of cannabis and risk of developing schizophrenia (Marconi et al., 2016; Urits et al., 2020; Volkow et al., 2016). At the individual level, this increased risk occurs in both sexes, but especially appears higher in young males. At the population level, this translates to CUD being a major modifiable risk factor for schizophrenia, particularly among males. Notably, an increasing proportion of cases with schizophrenia may be avertible by preventing CUD, and this increase is likely linked to the increase in THC concentration in cannabis as has been observed in confiscated samples in Denmark (Freeman et al., 2019; Thomsen et al., 2019). This apparent of schizophrenia conferred by CUD, in combination with observations that cannabis use among youth is associated with impaired cognition and reduced academic achievement (Lorenzetti, Hoch, & Hall, 2020), highlights the need to prevent cannabis use among youth and young adults. Interestingly, it has previously been shown that the association between cannabis and schizophrenia may be bidirectional (Ferdinand et al., 2005; Petersen, Toftdahl, Nordentoft, & Hjorthøj, 2019), and further investigation of the reverse association, schizophrenia being a risk factor for future cannabis use, by sex and over time warrants further study.

Our study has several strengths. The use of nationwide registers largely removes the risk of selection bias, since consent was not required for study sample participation. Furthermore, this data source reduces information bias to a certain degree, as a large range of information is available in the registers. Moreover, the registers are free from missing data in the traditional sense. Our results are likely highly generalizable to populations exposed to the same types of cannabis as are available on the Danish market. Finally, the national register-based nature of the longitudinal data over 5 decades allowed us to study nearly six million people, providing highly robust risk estimates.

This study also has certain limitations. The register-based nature of the study means that we only have information on both diagnosed CUD and diagnosed schizophrenia. This bias, however, is likely to be toward the null hypothesis, thus indicating that our results may be conservative, and PARFs of CUD on schizophrenia may be underestimated. Moreover, unmeasured and residual confounding factors likely exist, as the registers do not provide information on potentially important items such as frequency and amount of cannabis used, age of first use, or the THC content of cannabis products. Furthermore, we did not have access to genetic information, but as mentioned above, genetic confounding factors are unlikely to account for the observed differences. Finally, although the observational nature of this study does not directly allow for causal inference and we cannot be certain of the proportion of exposed individuals who might have developed schizophrenia even in the absence of CUD, it is unlikely that all of the associations between CUD and schizophrenia would be explained by confounding factors [e.g. tobacco use disorder (Fergusson et al., 2015; Gurillo et al., 2015)].

A further limitation is that the contents of the registers change over time. For instance, prior to 1995, outpatient psychiatric contacts were not included in the registers. Based on the results of our joinpoint regression analyses, there was no significant joinpoint identified in 1995 or years after 1995. It is thus unlikely to influence the increase in PARFs which only occurred later. We adjusted our models for other psychiatric disorders, which was the driver of the dramatic reduction in the magnitude of the HR compared to the unadjusted models. This is likely a case of over-adjustment, as some of these other psychiatric disorders might well be intermediate diagnoses between CUD and schizophrenia, and thus act as mediators rather than confounders. Consequently, our estimated aHR are likely highly conservative. Finally, we decided not to adjust for socioeconomic status, as this is more likely to be a product of CUD, other alcohol or substance use disorders, or schizophrenia, and thus not a potential confounder. By contrast, one of the salient factors is having a family history of schizophrenia. We controlled for the parental history of schizophrenia in our analyses. Moreover, we adjusted for age, sex, AUD, other substance use disorders, other psychiatric disorders, and parental history of CUD, AUD, and other psychiatric disorders, which are highly associated with schizophrenia.

In conclusion, this study finds strong evidence of an association between CUD and schizophrenia among both males and females, and the magnitude of this association appears to be consistently larger among males than females, especially among those aged 16–25. Importantly, 15% of cases of schizophrenia in males may be preventable if CUD was avoided. Although CUD is not responsible for most schizophrenia cases in Denmark, it appears to contribute to a non-negligible and steadily increasing proportion over the past five decades. In young males (21–30 years, possibly up to 40), the proportion may even be as high as 25–30%. There are global increases in legalization of nonmedical use of cannabis, increases in THC content of cannabis and in total THC doses consumed (Caulkins, Pardo, & Kilmer, 2020), increases in the prevalence of cannabis use and CUD, and decrease in public perception of harm from cannabis use (Chiu, Hall, Chan, Hides, & Leung, 2022). Alongside the increasing evidence that CUD is a modifiable risk factor for schizophrenia, our findings underscore the importance of evidence-based strategies to regulate cannabis use and to effectively prevent, screen for, and treat CUD as well as schizophrenia.

## Supporting information

Hjorthøj et al. supplementary material 1 Hjorthøj et al. supplementary material

Hjorthøj et al. supplementary material 2 Hjorthøj et al. supplementary material

Hjorthøj et al. supplementary material 3 Hjorthøj et al. supplementary material

Hjorthøj et al. supplementary material 4 Hjorthøj et al. supplementary material

Hjorthøj et al. supplementary material 5 Hjorthøj et al. supplementary material

Hjorthøj et al. supplementary material 6 Hjorthøj et al. supplementary material

## Supplementary material

The supplementary material for this article can be found at https://doi.org/10.1017/S0033291723000880.
