# Supplementary figures and images for: Association between cannabis use disorder and schizophrenia stronger in young males than in females
Source: Psychol Med. 2023 May 4;53(15):7322–8. doi: 10.1017/S0033291723000880 (PMC10719679; doi:10.1017/S0033291723000880)

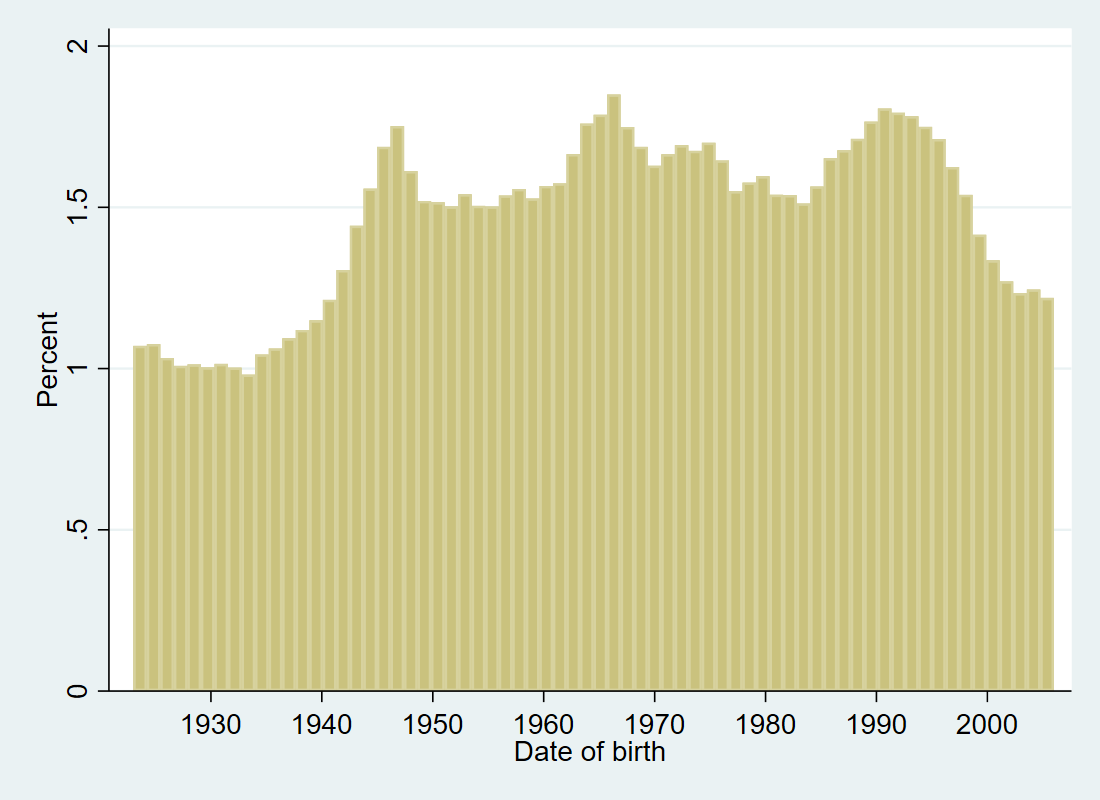

Supplement: Hjorthøj et al. supplementary material 1 — Hjorthøj et al. supplementary material [file S0033291723000880sup001.tif]

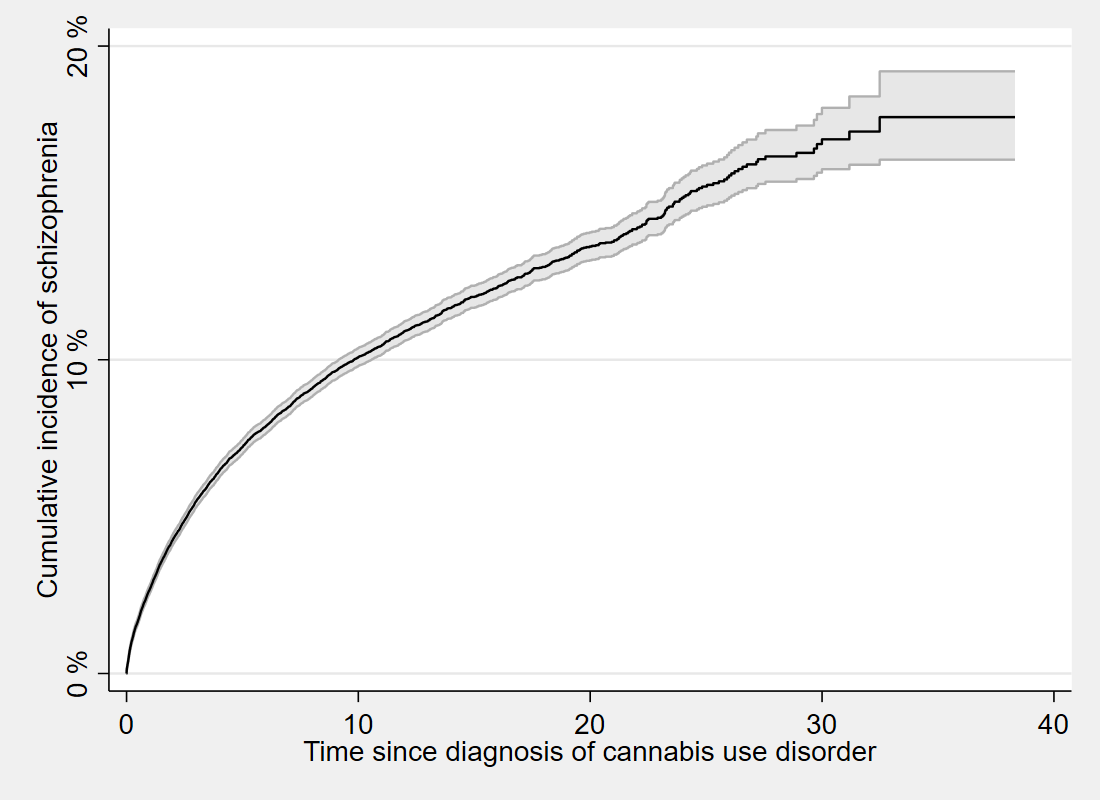

Supplement: Hjorthøj et al. supplementary material 2 — Hjorthøj et al. supplementary material [file S0033291723000880sup002.tif]

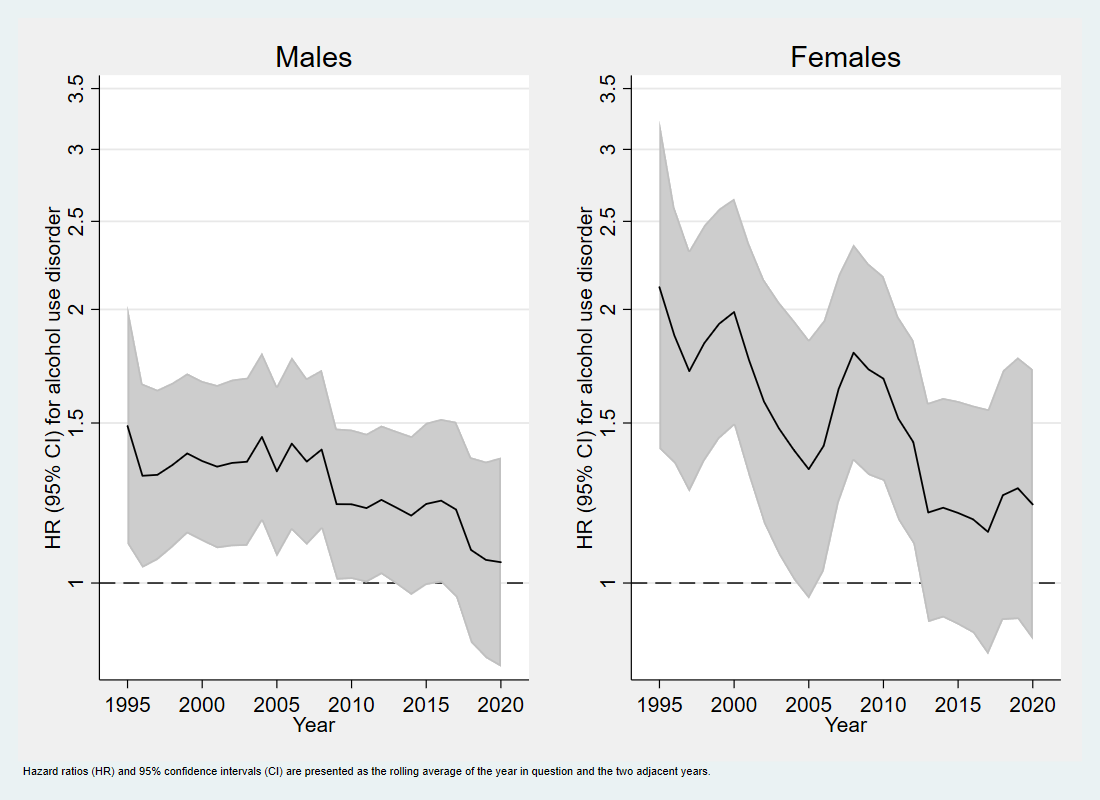

Supplement: Hjorthøj et al. supplementary material 3 — Hjorthøj et al. supplementary material [file S0033291723000880sup003.tif]

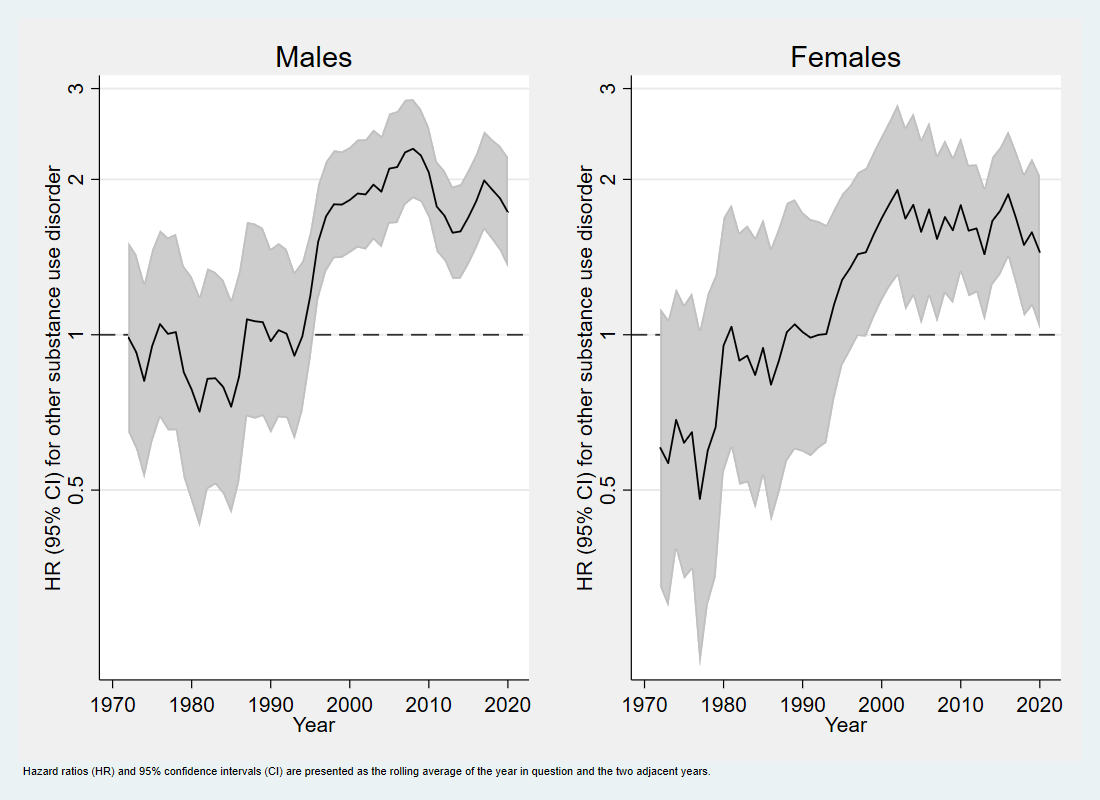

Supplement: Hjorthøj et al. supplementary material 4 — Hjorthøj et al. supplementary material [file S0033291723000880sup004.tif]

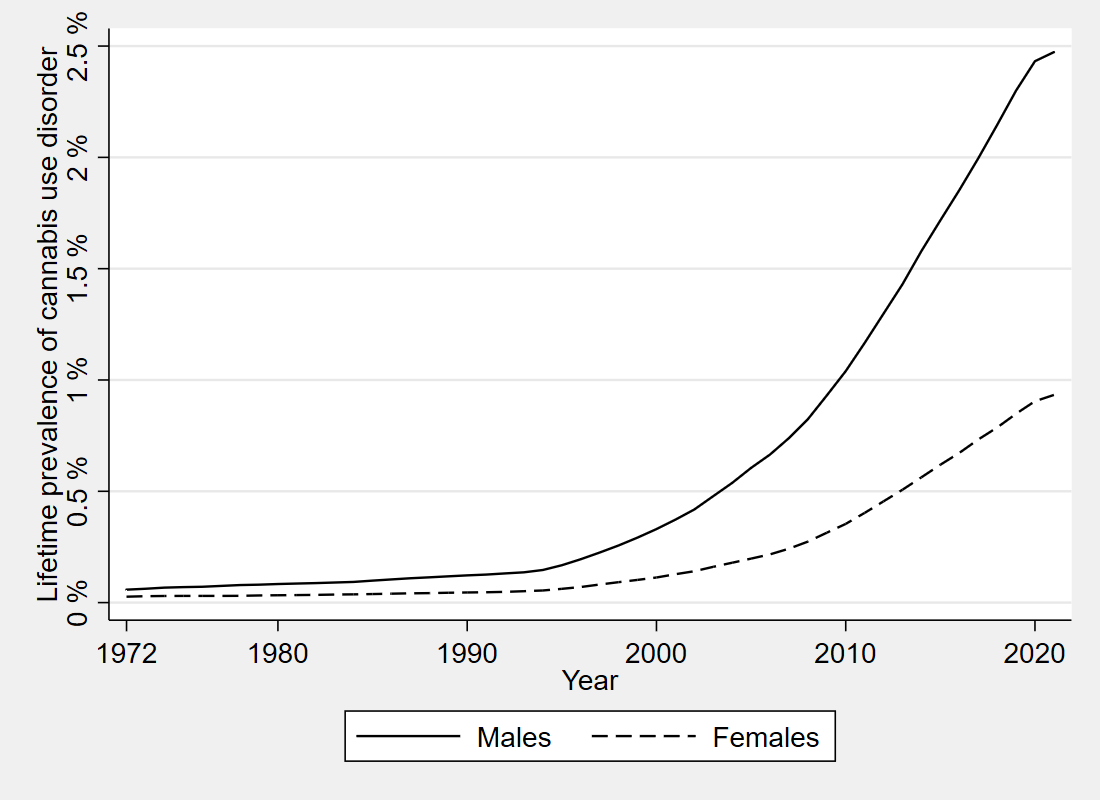

Supplement: Hjorthøj et al. supplementary material 5 — Hjorthøj et al. supplementary material [file S0033291723000880sup005.tif]
